# Chimeric Antigen Receptors Based on Low Affinity Mutants of FcεRI Re-direct T Cell Specificity to Cells Expressing Membrane IgE

**DOI:** 10.3389/fimmu.2018.02231

**Published:** 2018-10-10

**Authors:** Dana E. Ward, Brittany L. Fay, Adebomi Adejuwon, Huihui Han, Zhengyu Ma

**Affiliations:** ^1^Department of Biomedical Research, Nemours/A.I. duPont Hospital for Children, Wilmington, DE, United States; ^2^Department of Biological Sciences, University of Delaware, Newark, DE, United States

**Keywords:** adoptive T cell therapy, allergy, B cells, chimeric antigen receptor, IgE, allergic asthma

## Abstract

IgE is the key mediator of allergic responses. Omalizumab, an IgE-specific monoclonal antibody that depletes IgE, is effective for treating severe allergic asthma. The need for frequent administration of the expensive drug, however, limits its applications. Taking advantage of T cell memory, adoptive T cell therapy (ACT) targeting IgE-producing cells has the potential to achieve long-term suppression of IgE and relief of symptoms for severe allergic diseases. The transmembrane form of IgE (mIgE), which is present on all IgE-producing cells, serves as an excellent molecular target for ACT that employs chimeric antigen receptors (CARs). Here, we designed and tested CARs that use the extracellular domain of high affinity IgE receptor, FcεRIα, for mIgE recognition. When expressed on Jurkat T cells, FcεRIα-based CARs mediated robust responses in terms of CD69 upregulation to U266 myeloma cells expressing low levels of mIgE. FcεRIα-based CARs specifically recognized cells expressing mIgE, but not cells with secreted IgE captured through Fcε receptors. CAR^+^ Jurkat cells did not respond to LAD2 mast cells with secreted IgE bound through FcεRI or Ramos cells with secreted IgE bound through FcεRII. Co-culture of CAR^+^ Jurkat cells and LAD2 mast cells with IgE bound did not trigger LAD2 cell degranulation. The activity of CAR using wild type FcεRIα for mIgE binding was inhibited by the presence secreted IgE, which likely blocked CAR-mIgE interaction. The activities of CARs using low affinity mutants of FcεRIα, however, tolerated secreted IgE at relatively high concentrations. Moreover, primary human CD8^+^ T cells expressing a low affinity mutant CAR responded to U266 cells with INFγ production and cytotoxicity despite the presence of secreted IgE. The potency, specificity, and robustness of our CAR design, combined with repaid advances in the safety of ACT, hold promise for novel and highly effective cell-based therapies against severe allergic diseases.

## Introduction

In recent decades, the prevalence of allergic diseases has increased rapidly in developed countries, with more than 30% of children allergic, up to 10% of children having asthma and allergic rhinitis, and 5–7% of children having food allergies (1). Certain severe allergic diseases, such as severe allergic asthma and multiple food allergy, significantly impact quality of life, create heavy social and economic burdens, and cannot be effectively managed with currently available medications. IgE-mediated immune responses are central to the pathogenesis of allergy. IgE antibodies bind to the high affinity IgE receptor FcεRI expressed on mast cells, eosinophils, and basophils. Cross-linking of IgE and FcεRI by allergens triggers the degranulation and release of inflammatory mediators that induce type I hypersensitivity reactions and allergic symptoms. IgE is therefore an attractive target for therapeutic intervention. The effectiveness of omalizumab, an IgE-specific monoclonal antibody that depletes IgE, in treating severe allergic asthma clearly demonstrates the virtue of IgE targeting (2, 3). Omalizumab, however, has a relatively short half-life of 1–4 weeks (4). The need for repeated administration of the expensive drug limits its range of application. An approach that can persistently suppress IgE level over long term with a single treatment would therefore be highly desirable.

Targeting IgE-expressing cells, the source of IgE, using adoptive T cell therapy (ACT) has the potential to achieve long-term suppression of IgE. In ACT, autologous T cells are isolated and engrafted with engineered receptors that are specific for molecular markers on target cells. The cells are then infused back to patients to seek and destroy target cells. ACT using chimeric antigen receptors (CARs) specific for the pan-B cell marker CD19 have generated striking evidence of potent and long-lasting anti-cancer activity in humans (5–9), leading to its recent FDA approvals for B cell leukemia and lymphoma. Importantly, genetically modified T cells have been shown to establish memory (10) and persist for more than a decade in humans without adverse effects (11). Therefore, when applied to IgE-expressing B cells, ACT may achieve long-term relief of allergy symptoms, or even a cure of the disease, with a single treatment. By taking advantage of rapid advances in manufacturing processes and enhanced safety features, ACT may become increasingly attractive for severe atopic diseases such as severe allergic asthma, chronic urticaria and food allergies.

**Figure 1:**
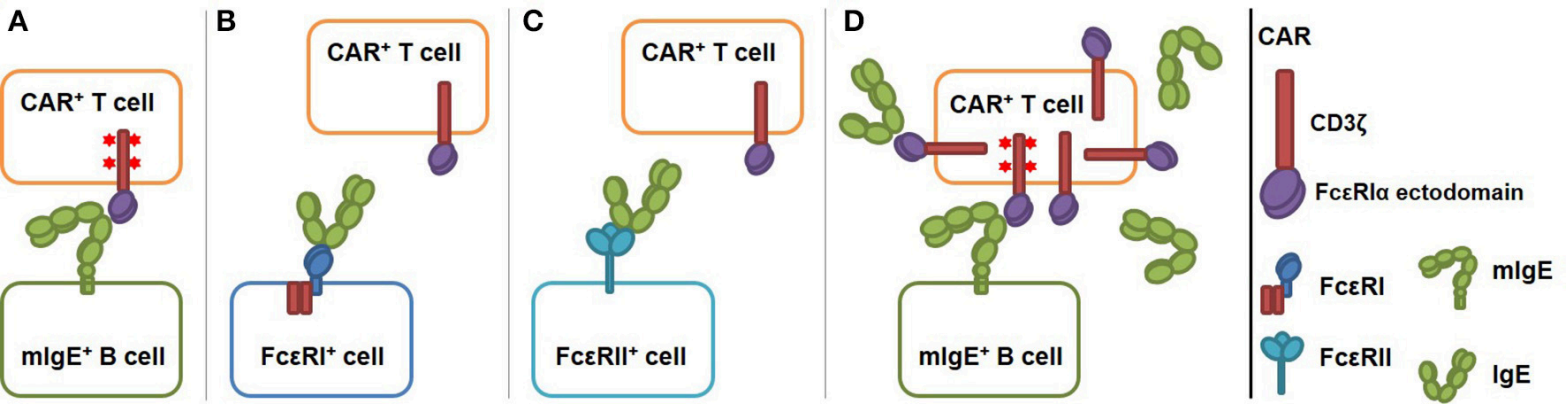
Schematics of low affinity FcεRIα-based CAR redirecting T cell responses specifically to cells expressing mIgE. The CAR is constructed by fusing the FcεRIα extracellular domain to the N-terminus of CD3ζ. **(A)** FcεRIα-based CARs on a T cell bind mIgE and mediate T cell responses to mIgE^+^ cells. IgE heavy chain assumes a bent conformation (14, 15) and the binding site for FcεRIα is at the bend (16). This confirmation should facilitate the engagement of mIgE by CARs on T cells. The binding triggers TCR signaling (shown as red stars at the signaling domains), T cell activation and target cell killing. **(B)** The CAR should not recognize cells with IgE captured through FcεRI since IgE has only one FcεRI binding site. **(C)** The CAR should not recognize cells with IgE captured through FcεRII because IgE-FcεRII binding allosterically inhibits IgE-FcεRI binding. **(D)** Low affinity FcεRIα-based CAR should tolerate the presence of secreted IgE. Low affinity CARs on T cells are only partially blocked by secreted IgE. Unoccupied CARs should still able to recognize mIgE on target cells as in **(A)**.

IgE-expressing cells can be targeted by T cells through the recognition of transmembrane form of IgE (mIgE). mIgE is expressed exclusively on all IgE-expressing cells, including germinal center B cells, plasmablasts, plasma cells, and memory B cells (12, 13). To this end, we designed and tested CARs that use the extracellular domain of FcεRI α chain (FcεRIα) for mIgE binding (Figure 1A). FcεRI consists of an α chain (FcεRIα) that binds to IgE Fc region with high affinity (K_d_ ≈3.7 × 10^−10^ M), and β and γ chains with intracellular signaling domains. Unlike FcεRII (CD23), which binds to MHC class II, integrins and CD21 in addition to IgE (17), FcεRI is known to only bind IgE at the Cε3 domain. Since Cε3 domain exists on both mIgE and secreted IgE, FcεRIα-based CAR must avoid targeting cells with secreted IgE captured by FcεRI or FcεRII (Figures 1B,C). As illustrated in Figure 1B, because IgE has only one FcεRIα binding site, the CAR should not recognize secreted IgE already bound to FcεRI on mast cells, eosinophils, basophils, and Langerhans cells (16, 18, 19) and trigger their killing or activation. Although FcεRI and FcεRII bind IgE at two distinct sites on the Cε3 domain, FcεRII-IgE binding allosterically inhibits FcεRI-IgE binding (20). Therefore, the CAR should not recognize secreted IgE bound to FcεRII on B cells and other cell types (21, 22) (Figure 1C). An issue associated with the high affinity of FcεRI-IgE binding is that FcεRIα-based CARs on T cells may be blocked by secreted IgE in circulation and tissues, rendering them unable to interact with mIgE on target cells (Figure 1D). To address this issue, we designed CARs using FcεRIα mutants with lower affinities for IgE. We reason that at a given concentration of secreted IgE, a T cell expressing low affinity CARs should have a smaller proportion of CARs bound (blocked) by secreted IgE than a cell expressing high affinity CARs. The relative low affinity of the CARs, however, should not affect their abilities to mediate T cell activation through mIgE binding, since CARs that bind ligands with K_d_ from 10^−6^ to 10^−9^ M have been shown to function effectively (23). Here, we demonstrated that low affinity FcεRIα-based CARs are capable of mediating potent and specific T cell responses to mIgE-expressing target cells in the presence of secreted IgE.

## Materials and methods

### Cells and antibodies

Jurkat (clone E6-1) acute T cell leukemia cells, U266 multiple myeloma cells, Ramos Burkitt's lymphoma cells (clone 2G6.4C10), and Daudi Burkitt's lymphoma cells were purchased from ATCC and cultured in complete RPMI medium containing 10% heat-inactivated FBS, 100 U/mL penicillin, 100 mg/mL streptomycin sulfate, and 2 mM L-glutamine. Daudi cells expressing human mIgE (Daudi-mIgE) were obtained from Genentech (24). LAD2 human mast cells were kindly provided by Dr. Metcalfe (NIH) (25) and cultured in complete StemPro-34 serum free medium (Invitrogen) supplemented with 100 ng/ml of stem cell factor (SCF) (Peprotech). Primary human CD8^+^ T cells were obtained from the Human Immunology Core at the University of Pennsylvania and cultured in complete RPMI medium. Fluorescently labeled antibodies for human FcεRIα, FcεRII (CD23), CD69, CD117, and human IgE were from Biolegend. Purified human IgE was purchased from Abcam and was further purified through gel filtration chromatography using a Superdex 200 column to eliminate aggregates. For degranulation assays, IgE was biotinylated using NHS-PEG4-bio (Pierce) following the manufacturer's instruction and purified using a Zeba desalting column (Pierce).

### Car design and construction

Human FcεRIα cDNA was PCR amplified from plasmid pcDL-huFcεRIα, a gift from Kochan et al. (26) (Addgene plasmid # 8365). Human CD3ζ cDNA was cloned from Jurkat cells using RT-PCR. The wilde type (WT) CAR was constructed by fusing the FcεRIα signaling peptide and extracellular domain to the N-terminus of human CD3ζ using overlapping PCR. The FcεRIα extracellular domain was mutated using PCR to generate six low affinity mutants: M1 (K117D), M2 (K117D+D159A), M3 (K117D+Y131A), M4 (K117D+W113A), M5 (K117D+W87D) (Table 1).

**Table 1:** The mutations and affinities of mutant CARs.

**CAR designation**	**Mutation (fold affinity reduction)**
M1	K117D (27x)
M2	K117D (27x) and D159A (2x)
M3	K117D (27x) and Y131A (3x)
M4	K117D (27x) and W113A (5x)
M5	K117D (27x) and W87D (7x)
M6	K117D (27x) and V155A (10x)

### Lentiviral transduction of jurkat T cells and primary human T cells

DNA encoding CARs were inserted into the pLVX-EF1α-IRES-Puro lentiviral transfer vector (Clontech). Lentiviral vectors were packaged using 293T cells with the Lenti-X packaging system (Clontech) and concentrated by ultrafiltration using Centricon Plus-70 filters (EMD Millipore) at 1,500 rpm for 2 h at 15°C. Jurkat T cells were transduced with lentiviral vectors using spinoculation in a 48-well tissue culture plate. The plate was spun at 2,500 rpm for 90 min at 32°C. The transduced cells were selected in complete RPMI medium containing 0.25 μg/ml puromycin. To transduce primary human CD8^+^ T cells, T cells were stimulated with human T-activator CD3/CD28 Dynabeads (Life Technologies) at a 3:1 bead to cell ratio for 1 day, followed by spinoculation in the presence of 10 μg/ml protamine sulfate (Sigma-Aldrich). Beads were removed 2 days later, and the cells were cultured and expanded in complete RPMI medium containing 300 IU/ml recombinant human IL2 (R&D Systems) for 3–5 additional days before being used for experiments.

### Jurkat cell stimulation

To use Ramos cells for stimulation, FcεRII expression was upregulated by stimulating with 20 ng/ml human IL4 (Peprotech) for 72 h. To bind IgE to Ramos cells and LAD2 cells, the cells were incubated with IgE for 1 h on ice, and IgE binding was confirmed with flow cytometry using IgE-specific antibodies. For Jurkat cell stimulation, 0.125 × 10^6^ Jurkat cells were mixed with equal numbers of U266 cells, Ramos cells with IgE bound, or LAD2 cells with IgE bound in 200 μl of medium, incubated for 5 h at 37°C, and stained with anti-CD69 antibodies for flow cytometry analysis. To distinguish CAR^+^ Jurkat cells from stimulator cells, in addition to anti-CD69-APC, anti-FcεRI-PE was used for co-culture with U266 or Ramos cells to label CAR^+^ Jurkat cells. Since LAD2 cells express FcεRI, to separate LAD2 cells and CAR^+^ Jurkat cells in co-cultures, anti-CD117-PE was used to label LAD2 cells. To distinguish Daudi or Daudi-IgE cells with Jurkat cells in co-cultures, Jurkat cells were first labeled with the intracellular fluorescent dye carboxyfluorescein diacetate succinimidyl ester (CFSE) by incubating with 0.3 μM CFSE in DPBS-5% FBS for 5 min at room temperature (27).

### LAD2 cell degranulation assay

The assay was performed as described in a previously published protocol (28) with minor modifications. Briefly, LAD2 cells were cultured in complete RPMI medium containing 0.2 μg/ml biotinylated IgE overnight. After washing with assay buffer (DPBS containing 10 mM HEPES, 5.6 mM glucose, and 0.04% BSA, pH 7.4), 1 × 10^4^ LAD2 cells were transferred to a 96-well plate and mixed with 2 × 10^5^ CAR^+^ Jurkat cells in assay buffer. For positive controls, streptavidin was added to a final concentration of 1 μg/ml. Cells were incubated at 37°C for 30 min and spun at 450 × g for 5 min at 4°C. The supernatant was removed, and the cells were lysed with 0.1% Triton X-100 in assay buffer. β-hexosaminidase activities in the supernatant and lysate were determined using N-acetyle-β-D-glucosamide (PNAG) (Sigma) as substrate. The low levels of β-hexosaminidase activity from Jurkat cells were determined in control samples with Jurkat cells alone and subtracted from the assay results. The percentage degranulation was calculated as 100x (supernatant activity)/(supernatant activity + lysate activity).

### Primary T cell activation and luciferase-based cytotoxicity assay

To generate target cells for luciferase-based cytotoxicity assay (29, 30), U266 cells were transduced with pLVX-EF1α-IRES-Puro lentiviral vectors encoding firefly luciferase and selected in complete RPMI medium supplemented with 0.5 μg/ml puromycin for stable expression. Since U266 cells express high levels of MHC class I (31), to inhibit allo-activation of primary T cells, human CD8^+^ T cells were incubated with 10 μg/ml anti-CD8 antibody (clone SK1) for 30 min before stimulation. SK1 has been shown to effectively block T cell activation through T cell receptor (TCR)-MHC class I interaction, in which CD8 is critically involved (32, 33). T cells were then co-cultured with 5 × 10^4^ U266-luciferase cells for 16 h in complete RPMI medium containing 10 μg/ml SK1 antibody. INFγ levels in the supernatant were measured using a human INFγ ELISA kit (Biolegend). Luciferase activity in live U266-luciferase cells was determined using the Bright-Glo luciferase assay system (Promega) on a Victor X luminescence microplate reader (Perkin Elmer). The luciferase activity of 5 × 10^4^ U266-luciferase cells cultured without T cells was determined as maximum activity. Specific lysis was calculated as [1–(sample activity)/(max activity)] × 100.

### Flow cytometry

Cells (0.25 × 10^6^) were washed twice with FACS buffer (DPBS with 0.5% BSA and 0.02% sodium azide) and stained with fluorescently-labeled antibodies described above for 30 min on ice. Stained cells were washed twice with FACS buffer and analyzed on an Accuri C6 flow cytometer (BD Biosciences).

## Results

### FcεRIα-based CARs mediate potent T cell responses to migE^+^ Cells

To construct FcεRIα-based CARs, the extracellular domain of human FcεRIα is fused to the N-terminal of the human CD3ζ extracellular domain. The transmembrane domain of CD3ζ in this CAR design should facilitate its association with the endogenous TCR/CD3 complex, thereby enhancing CAR sensitivity (34). To create CARs with lower affinities for IgE, we introduced point mutations to FcεRIα that were shown to reduce its binding affinity for IgE in previous mutagenesis studies (35, 36). The first mutant CAR (M1) has a single K117D mutation that has been shown to reduce affinity by 27-fold (35). Five other mutant CARs (M2 to M6) each included an additional mutation that has been shown to reduce the affinity to varying degrees (36) (Table 1). Although the exact affinities of the mutant CARs with two point mutations are unknown, the wild type (WT) and six mutant CARs should represent a relatively wide range of affinities for IgE binding.

**Figure 2:**
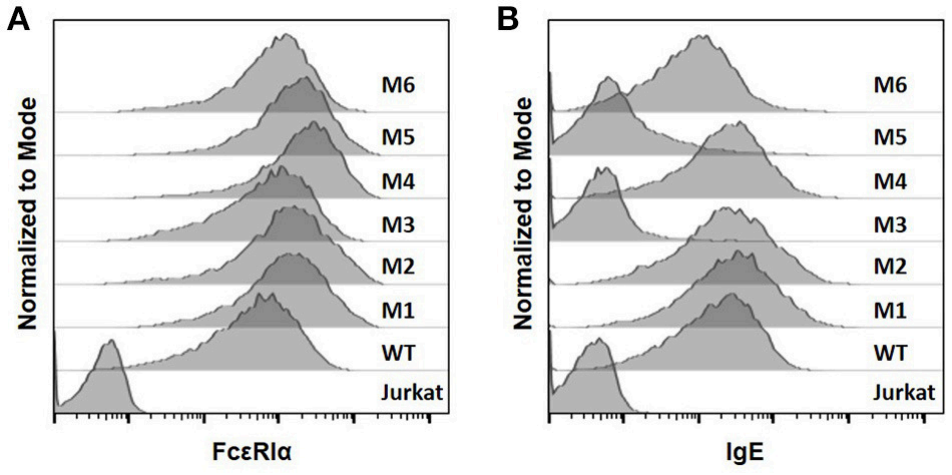
The expression of FcεRIα-based CARs on Jurkat T cells and IgE binding. **(A)** Jurkat cells stably transduced with lentiviral vectors encoding WT and mutant CARs were stained with anti-FcεRIα-PE antibody and analyzed by flow cytometry. Un-transduced Jurkat cells served as negative control. **(B)** Jurkat cells in **(A)** were incubated with 10 μg/ml human IgE on ice for 1 h, washed, and stained with anti-IgE-APC antibody. Data are representative of at least three independent experiments.

**Figure 3:**
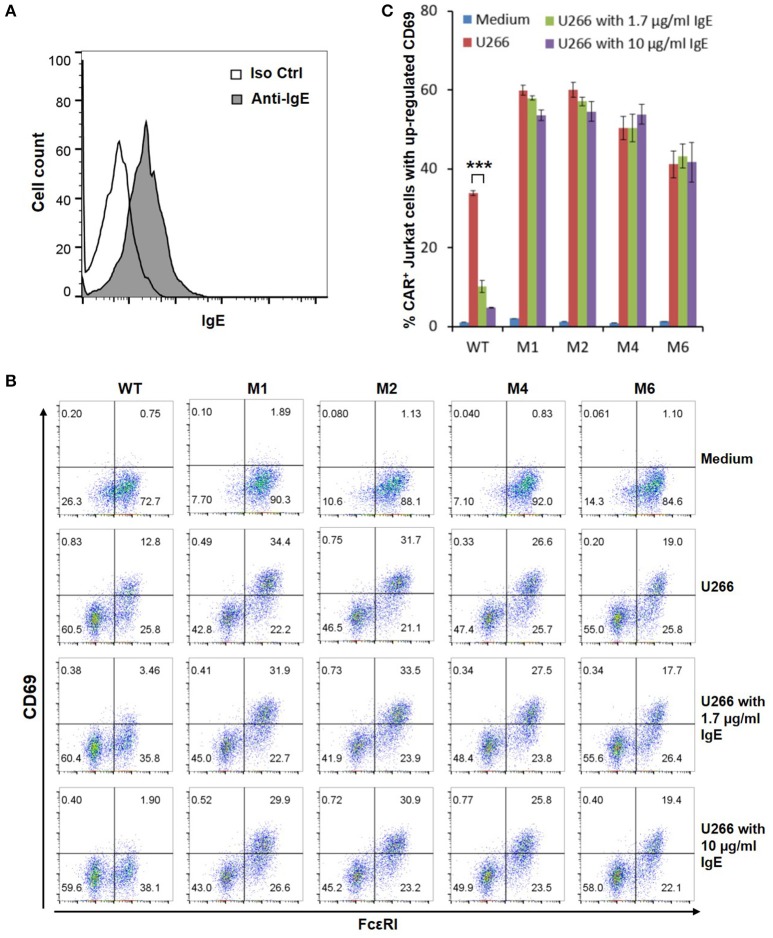
Low affinity FcεRIα-based CARs mediate specific T cell responses to mIgE^+^ U266 cells in the presence of secreted IgE. **(A)** U266 cells stained with anti-IgE antibody showed low levels of mIgE expression. **(B)** Jurkat cells expressing the WT and mutant CARs were stimulated with U266 cells in the absence or presence of secreted IgE at 1.7 or 10 μg/ml for 5 h and stained with anti-FcεRI and anti-CD69 antibodies. **(C)** Calculated percentages of CAR^+^ T cells with upregulated CD69 in response to U266 stimulation as shown in **(B)**. Data are presented as mean ± SD (*n* = 3). ***P < 0.001, unpaired *t-*test.

**Figure 4:**
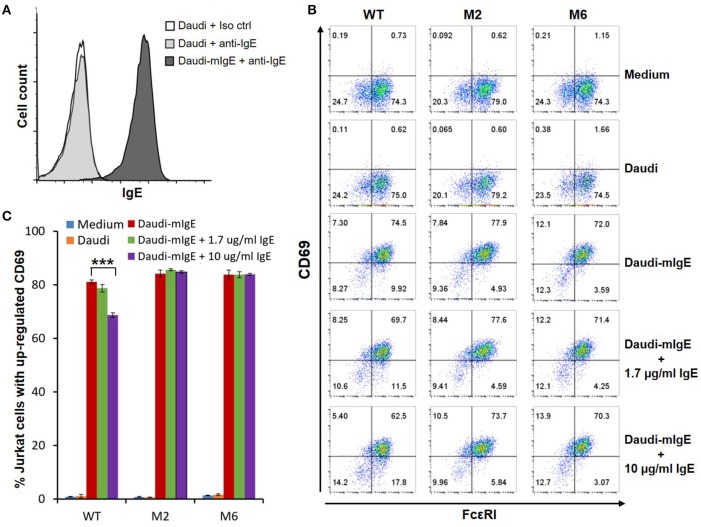
Low affinity FcεRIα-based CARs mediate specific T cell responses to Daudi-cells expressing high levels of mIgE in the presence of secreted IgE. **(A)** Daudi-mIgE cells stained with anti-IgE antibody showing high levels of mIgE expression. **(B)** Jurkat cells expressing the WT, M2, and M6 CARs were pulsed with CFSE and cultured with Daudi or Daudi-mIgE cells in the absence or presence of secreted IgE at 1.7 or 10 μg/ml for 5 h. The cells were harvested and stained with anti-FcεRI and anti-CD69 antibodies. CAR and CD69 expression levels are shown for CFSE^+^ Jurkat cells. **(C)** Calculated percentages of total Jurkat cells with upregulated CD69 as shown in **(B)**. Data are presented as mean ± SD (*n* = 3). ***P < 0.001, unpaired *t-*test.

The WT and six mutant CARs were expressed on human Jurkat T cells at generally comparable levels through lentiviral transduction, and stable expressers were selected with puromycin (Figure 2A). Jurkat cells expressing WT, M1, M2, M4, and M6 CARs were able to bind IgE, and the levels of binding largely mirrored levels of CAR expression (Figure 2B). Jurkat cells expressing M3 and M5, however, showed very low levels of IgE binding (Figure 2B). The Y131A and W87D mutations most likely worked synergistically with K117D to dramatically reduce FcεRIα binding to IgE. As expected, CAR^+^ Jurkat cells were not activated by secreted IgE (Supplementary Figure 1). To determine whether the WT and mutant CARs can mediate T cell responses to cells expressing mIgE, CAR^+^ Jurkat cells were stimulated with U266 cells, a human myeloma line expressing low levels of mIgE (Figure 3A). As shown in Figures 3B,C, Jurkat cells expressing WT, M1, M2, M4, and M6 CARs showed marked up-regulation of CD69, a T cell activation marker. CD69 up-regulation mediated by the WT CAR was significantly lower than those by mutant CARs. M1 and M2 CARs showed the strongest activity, followed by M4 and M6 CARs. The lower activity of the WT CAR may be attributable to its relatively low expression level (Figure 2). To assess the CARs' ability to mediate responses to target cells expressing high levels of mIgE, Daudi cells stably expressing human mIgE (Figure 4A) were used to stimulate Jurkat cells expressing the WT, M2, and M6 CARs. The Jurkat cells responded to Daudi-mIgE stimulation with similar levels of robust CD69 upregulation (Figures 4B,C). As expected, Jurkat cells expressing M3 and M5 did not show significant responses to U266 or Daudi-mIgE stimulation (Supplementary Figure 2).Taken together, these results demonstrated that WT and low affinity FcεRIα-based CARs are capable of mediating robust T cell responses to mIgE^+^ target cells.

### T cell responses mediated by low affinity FcεRIα-Based CARs tolerate secreted IgE

Under physiological conditions, secreted IgE present in tissues may bind to FcεRIα-based CARs and block their interaction with mIgE on target cells. To test the effects of secreted IgE on CAR function, CAR^+^ Jurkat cells were stimulated with U266 cells in the presence of IgE at 1.7 μg/ml and 10 μg/ml. A concentration of 1.7 μg/ml is equivalent to 700 IU/ml, the upper limit of serum IgE level recommended for omalizumab. As shown in Figures 3B,C, when stimulated by U266 cells, 1.7 μg/ml IgE suppressed CD69 upregulation of Jurkat cells expressing WT CAR by more than two-folds. Increasing IgE concentration to 10 μg/ml further suppressed CD69 upregulation. In contrast, the activities of low affinity M1, M2, M4, and M6 CARs were not significantly affected by the presence of IgE at 1.7 μg/ml or 10 μg/ml. In comparison, when stimulated by Daudi-mIgE, CD69 upregulation by Jurkat cells expressing the WT CAR was inhibited by secreted IgE at 10 μg/ml, but not at 1.7 μg/ml (Figures 4B,C). These results support our model that low affinity FcεRIα-based CARs tolerate the presence of secreted IgE at high concentrations (Figure 1D) and suggest that ACT employing low affinity FcεRIα-based CARs may be effective for patients with serum IgE levels higher than the limit for omalizumab.

### FcεRIα-Based CARs do not recognize cells with secreted IgE captured through FcεRII

**Figure 5:**
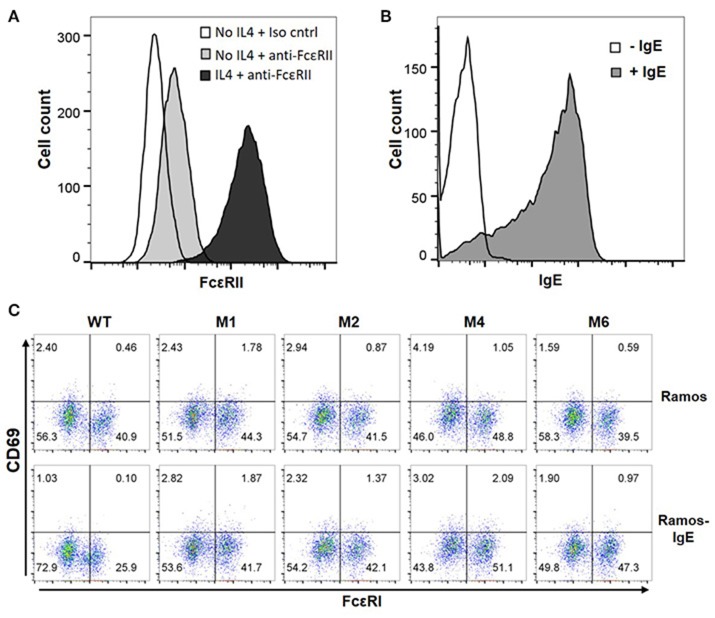
FcεRI-based CARs do not stimulate T cell responses to Ramos cells with secreted IgE captured through FcεRII. **(A)** Ramos cells upregulated FcεRII expression after stimulation with 20 ng/ml IL4 for 72 h. **(B)** Ramos cells stimulated with IL4 in **(A)** were incubated with 1.7 μg/ml IgE for 1 h on ice, washed, and stained with anti-IgE-APC antibody. **(C)** Jurkat cells expressing WT and mutant CARs were stimulated with Ramos cells or Ramos cells with secreted IgE captured through FcεRII for 5 h and stained for CD69 expression. Data are representative of three independent experiments.

The majority of B cells express FcεRII, and its binding to IgE regulates IgE production (37). FcεRII is also expressed on a variety of inflammatory cells and epithelial cells. Recognition of secreted IgE bound to FcεRII by CARs would therefore lead to significant adverse effects. To test our hypothesis that FcεRIα-based CARs do not recognize IgE captured by FcεRII due to allosteric inhibition (Figure 1C), we used Ramos cells, a Burkitt lymphoma cell line, as target cells. Consistent with previous reports (38), Ramos cells significantly up-regulated FcεRII expression in response to IL4 stimulation (Figure 5A) and bound IgE at high levels (Figure 5B). IgE binding by Ramos cells is exclusively through FcεRII since FcεRI expression was not detected (Supplementary Figure 3). As shown in Figure 5C, Jurkat cells expressing WT, M1, M2, M4, or M6 CARs did not significantly upregulate CD69 in response to Ramos cells with high levels of IgE bound. FcεRIα-based CARs therefore do not recognize cells with secreted IgE captured through FcεRII.

### FcεRIα-Based CARs do not recognize cells with secreted IgE captured through FcεRI

**Figure 6:**
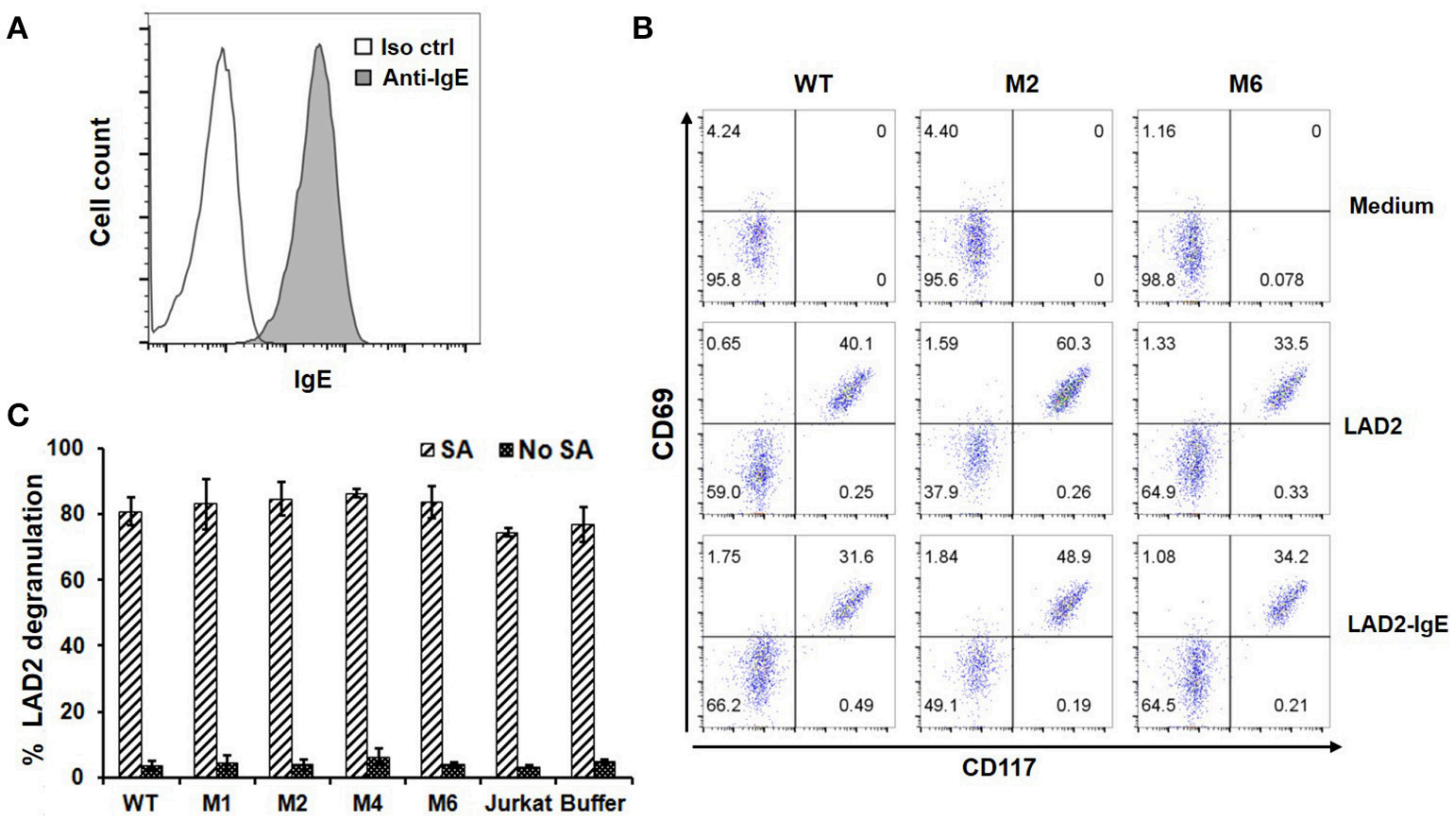
FcεRIα-based CARs do not mediate T cell responses to mast cells with free IgE captured through FcεRI, or trigger mast cell activation. **(A)** LAD2 cells were incubated with 1.7 μg/ml of IgE, and bound IgE was stained with anti-IgE antibody. **(B)** LAD2 cells with IgE bound were used to stimulate CAR^+^ Jurkat T cells for 5 h. Cells were collected and stained with antibodies for CD69 and CD117. CD117 is expressed only on LAD2 cells and therefore used to distinguish Jurkat and LAD2 cells. Note the high levels of constitutive CD69 expression by LAD2 cells. Data are representative of two independent experiments. **(C)** LAD2 cells were coated with biotinylated IgE at 1.7 μg/ml followed by incubation with CAR^+^ Jurkat T cells for 30 min in the presence or absence of streptavidin. The β-hexosaminidase activity was determined using PNAG as substrate, and percentage of degranulation was calculated. Data are presented as mean ± SD (*n* = 3).

Through high affinity interaction with FcεRI, secreted IgE may be stably bound to the surfaces of mast cells, eosinophils, basophils and Langerhans cells. Since IgE has only one binding site for FcεRI, we do not anticipate that FcεRIα-based CARs mediate cytotoxicity to these cells or trigger degranulation through CAR interaction with IgE bound to FcεRI (Figure 1B). To confirm this, LAD2 cells, a human mast cell line, were used as targets. LAD2 cells expressed FcεRI (Supplementary Figure 3) and bound secreted IgE at relatively high levels (Figure 6A). IgE binding by LAD2 cells is exclusively through FcεRI, as FcεRII expression was not detected (Supplementary Figure 3). As shown in Figure 6B and Supplementary Figure 4, Jurkat cells expressing WT, M1, M2, M4, or M6 CARs did not significantly upregulate CD69 in response to LAD2 cells with IgE bound. To determine whether CAR^+^ Jurkat cells can trigger degranulation of mast cells, LAD2 cells with biotinylated IgE bound were co-cultured with CAR^+^ Jurkat cells, control Jurkat cells, or buffer alone, in the presence or absence of streptavidin. LAD2 cell degranulation was then determined by measuring β-hexosaminidase release. As shown in Figure 6C, in the presence of streptavidin, which binds to biotinylated IgE and crosslinks FcεRI, high levels of degranulation (>70%) were induced in all co-cultures. In the absence of streptavidin, however, only background levels of degranulation were observed, indicating that FcRεI-based CARs are incapable of crosslinking the FcRεI-IgE complexes on LAD2 cells. Taken together, we conclude that FcεRIα-based CARs do not recognize cells with IgE captured through FcεRI.

### FcεRIα-Based M2 CAR directs primary human T cell responses to migE^+^ Target cells

**Figure 7:**
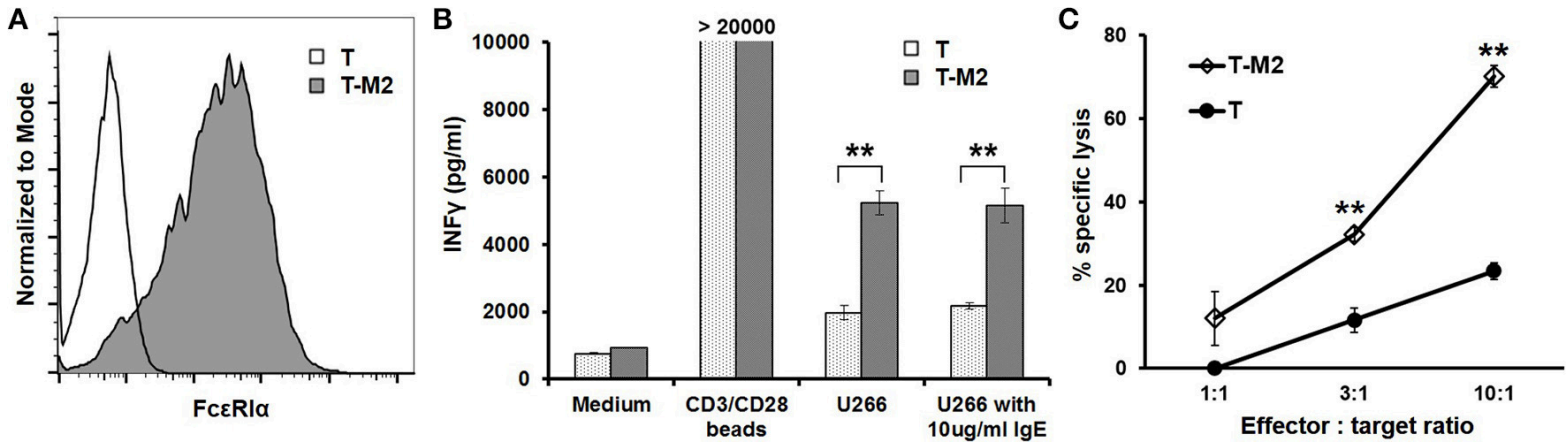
M2 CAR mediates potent primary human T cell responses to U266 cells. **(A)** Purified human CD8^+^ T cells were activated using anti-CD3/anti-CD28 beads and transduced with lentiviral vectors encoding the M2 CAR (T-M2). Cells were stained with anti-FcεRIα-PE antibody and analyzed by flow cytometry 7 days post-transduction. Untransduced human CD8^+^ T cells (T) served as negative control. **(B)** Human CD8^+^ T cells transduced with the M2 CAR (T-M2) or untransduced control T cells (T) were stimulated with equal numbers of CD3/CD28 beads, U266 cells, or U266 cells in the presence of 10 μg/ml IgE for 20 h. The concentrations of IFNγ in the supernatant were determined using ELISA. **(C)** CD8^+^ T cells expressing the M2 CAR (T-M2) or control T cells (T) were cultured with U266 cells stably expressing firefly luciferase at indicated ratios for 20 h. The percentages of specific lysis were determined based on the luciferase activity in the remaining live U266-luci cells. Data are presented as mean ± SD (*n* = 3). **P < 0.001, unpaired *t-*test.

To test the CAR function using primary T cells, we expressed the M2 CAR on primary human CD8^+^ T cells via lentiviral transduction (Figure 7A). The M2 CAR was chosen for its potency, specificity, and resistance to secreted IgE in mediating Jurkat cell responses to mIgE^+^ target cells (Figures 3–6). In response to U266 cells, M2 CAR^+^ T cells produced a significant amount of IFNγ (Figure 7B). Moreover, the activity of M2 CAR was not affected by the presence of 10 μg/ml of IgE. Finally, using a luciferase-based cytotoxicity assay (29, 30), we determined the cytotoxicity of M2 CAR^+^ primary human CD8^+^ T cells to U266 cells expressing luciferase. Consistent with the IFNγ data, the M2 CAR mediated the killing of U266-luciferase cells in a dose-dependent manner (Figure 7C). Taken together, the FcεRIα-based low affinity M2 CAR is capable of mediating potent primary T cell responses to mIgE^+^ target cells.

## Discussion

In this study, we designed and tested CARs that redirect T cell specificity to mIgE^+^ cells for ACT against allergic diseases. The FcεRIα-based CAR design takes advantage of the highly specific binding between FcεRI and IgE and the bent conformation of IgE that facilitates mIgE-CAR interaction at the T cell-target cell interface (14–16) (Figure 1). Compared with mouse-derived single chain variable fragments (scFvs) commonly used for antigen recognition by CARs, FcεRIα, a natural human protein, is unlikely to trigger host immune responses that may lead to low CAR T cell persistence or even systemic anaphylaxis (39). FcεRIα-based CARs mediated T cell responses to U266 myeloma cells expressing very low levels of mIgE (Figures 3, 7), suggesting that even low mIgE-expressing plasma cells may be targeted *in vivo*. Although the majority of IgE-producing plasma cells are short-lived, a small population of long-lived plasma cells in bone marrow may continuously produce IgE at low levels (12, 13, 40–43). The ability to eliminate plasma cells is therefore important for the long-term effectiveness of ACT against allergies. CAR-mediated Jurkat responses appeared to be more robust to Daudi-mIgE, a Daudi cell line engineered to stably express mIgE at high levels (Figure 4). In addition, the high levels of mIgE expression may have compensated the effects of lower WT and M6 expression levels observed in their relatively subdued responses to U266 (Figure 3), leading to similarly strong responses by WT, M2, and M6 to Daudi-mIgE (Figure 4). It is interesting that M1, M2, M4, and M6 CARs with at least 27-fold lower affinities are still capable of mediating strong T cell activation. This is consistent with the generally low affinity requirement for the antigen recognition by TCR (K_d_ ≈10^−6^ M) (44) and CAR (K_d_ ≈10^−6^−10^−9^ M) (23). The low affinity mutant CARs also outperformed the WT in tolerating the presence of secreted IgE at 10 μg/ml (Figures 3, 4, 7B), supporting our hypothesis that T cells expressing the WT CAR have a larger proportion of CARs blocked by secreted IgE than T cells expressing the low affinity mutants. From a kinetic point of view, the K117D mutation shared by all mutants causes a 145-fold increase in dissociation rate (k_off_) (35). The decreased stability should lead to frequent unbinding between CAR and secreted IgE and facilitate engagement of CARs by mIgE. Taken together, the low affinity mutant CARs, especially M1 and M2, may have the optimal affinity and k_off_ for sensitive mIgE recognition and tolerance of secreted IgE.

Using Ramos and LAD2 as target cells, we demonstrated that FcεRIα-based CARs do not recognize cells with secreted IgE bound through FcεRI or FcεRII (Figure 6). Sparing these cells is critical for avoiding side effects associated with their killing or activation in ACT. Targeting FcεRI-expressing mast cells, basophils and eosinophils, for example, would be especially problematic since it may trigger massive degranulation and anaphylaxis. It should be noted, however, that all potential issues associated with secreted IgE, including unwanted targeting of cells expressing FcεRI or FcεRII and blocking CAR function, can be ameliorated or eliminated by using omalizumab to decrease or deplete IgE prior to ACT. Preconditioning patients with omalizumab may therefore be employed in initial clinical studies for enhanced efficacy and safety.

The potency, specificity, and robustness of our CAR designs will aid the development of ACT for severe allergic diseases. The rapid advances in ACT's effectiveness, safety, and T cell manufacturing processes should make ACT for severe allergic diseases increasingly attractive. Compared with omalizumab, the main advantage of ACT is its potential for long-term symptom control. Achieving this goal critically relies on CAR T cell persistence. In this regard, the long-term persistence of anti-CD19 CAR T cells in patients with B cell malignancies is highly encouraging (10, 45). In addition to the pro-survival effects of CD28 or 4-1BB signaling domains, the persistence of anti-CD19 CAR T cells was thought to be boosted by repeated stimulation from continuously emerging B cells (46), which express co-stimulatory molecules. CAR T cells recognizing mIgE^+^ B cells should benefit from similar repeated stimulation from newly IgE class-switched B cells. Moreover, IgE-expressing B cells are highly concentrated in mucosal tissues (47, 48), which experience frequent inflammation events triggered by infection or allergy. The inflammatory milieu should attract infiltration of T cells, including CAR^+^ T cells, increase their chance of encountering IgE-expressing target cells, and enhance the development and maintenance of memory phenotype in a way that is similar to repeated respiratory tract viral infection. Finally, long term persistence may also be enhanced by adopting novel approaches such as expressing CARs on enriched virus-specific T cells (49) or *ex vivo* expanded central memory CD8^+^ T cells (50).

Using ACT to treat allergic diseases, even the severe forms, would require an improved understanding and control of risk factors associated with technologies currently used for cancer patients. The two main issues of current lentiviral vector-based CAR T cell technologies are the generation of replication competent viruses and oncogenesis associated with random gene insertion. So far, these risks are only theoretical since they have not materialized in patients enrolled in a large number of ACT clinical trials to date. Additional data from clinical application of the recently approved anti-CD19 CAR T cell therapies will help establish a more accurate safety profile of these approaches. The risk of replication competent viruses can be minimized by using packaging systems with advanced safety features such as the separation of packaging components into multiple plasmids and inclusion of self-inactivating (SIN) elements. The rapid development of non-viral gene integration through transposon/transposase systems should eliminate such risk (51). Regarding insertion-related oncogenesis, a longitudinal study showed that CAR^+^ T cells persisted for more than a decade in patients without causing oncogenesis-related adverse effects (11). This suggests that retroviral manipulation of mature T cells is fundamentally safe, likely because integration sites are not random and do not favor proto-oncogenes (52). The risk of insertion-related oncogenesis may be fully addressed by targeting CARs to a harmless location through genome editing (53, 54). For example, the anti-CD19 CAR was recently targeted to the TCRα locus through CRISPR/Cas9-based genome editing that used templates delivered with non-integrating adeno-associated viral vectors (53). Most recently, the endogenous TCR locus was efficiently replaced with a new TCR that recognizes cancer antigen though electroporation of T cells with CRISPR-Cas9 ribonucleoprotein complexes and linear double strand DNA templates (55). If reproducible, this non-viral targeted genome editing approach should dramatically reduce risks associated with both replication-competent viruses and insertion-associated oncogenesis. Finally, the risks of ACT may be further controlled through the incorporation of safety features to CAR T cells (56), such as incorporating an inducible caspase-9-based suicide mechanism (57) and employing CARs with built in on-switches that are active only when triggered by certain small molecule drugs (58).

A potential issue with long-term suppression of IgE-expressing B cells using ACT is that reduced IgE level may lead to increased incidents of parasitic infection or malignancy. Although IgE is capable of mediating parasite killing, its role in controlling parasitic infection has been debated (59–61). In addition, in a study of subjects at high risk of helminth infection, omazilumab was not associated with increased morbidity (59). The role of IgE in the immune surveillance of cancer is controversial. Moreover, a recent long-term study showed no increase in incidents of malignancy in patients treated with omalizumab (62). These findings suggest that long-term suppression of IgE using ACT should be relatively safe. Finally, our ACT approach targets only IgE-expressing B cells, which makes up a very small fraction of total B cells. In normal individuals, serum IgE concentration is 10,000–100,000 times lower than IgG (37). Our IgE-specific approach therefore should not significantly impact overall humoral immunity, which is mediated mostly by IgG antibodies.

Although the costs of recently approved CAR T cell therapies for B cell cancers are high, their pricing is based on both manufacturing costs and the high cost of bone marrow transplant-based conventional treatment regimens. The manufacturing costs can be expected to decrease over time with increasingly streamlined and automated processes and possibly the adoption of non-viral gene transfer approaches (55). Advances in developing “off-the-shelf” allogeneic T cell- or NK cell-based ACT (63, 64) may reduce costs dramatically. In addition, the much higher number of patients with severe allergic diseases than B cell cancers may justify lower prices. Therefore, it is possible that ACT may become competitive with omalizumab in overall cost if long-term effectiveness can be established.

In summary, FcεRIα-based CARs mediate potent and specific T cell responses to mIgE^+^ target cells. Future studies on the activity and persistence of T cells expressing the CARs *in vivo* may lead to the development of ACT with long-term effectiveness for severe allergic diseases.

## Author contributions

DW performed most of the experiments and collected data. BF, AA, and HH performed experiments, collected data, and contributed to the writing of the manuscript. ZM designed the study, carried out experiments, analyzed the data, and wrote the manuscript.

### Conflict of interest statement

ZM holds a patent (pending) on using FcεRI-based CARs for allergic diseases. The remaining authors declare that the research was conducted in the absence of any commercial or financial relationships that could be construed as a potential conflict of interest.
