# Supplementary material for: Chimeric Antigen Receptors Based on Low Affinity Mutants of FcεRI Re-direct T Cell Specificity to Cells Expressing Membrane IgE
Source: Front Immunol. 2018 Oct 10;9:2231. doi: 10.3389/fimmu.2018.02231 (PMC6191488; doi:10.3389/fimmu.2018.02231)
Supplement: Supplementary file 1 [file Data_Sheet_1.pdf]

Supplementary Material

Chimeric antigen receptors based on low affinity mutants of FcεRI re-direct T cell specificity to cells expressing membrane IgE

Dana E. Ward[1], Brittany L. Fay[1], Adebomi Adejuwon[2], Huihui Han[1], Zhengyu Ma[1]*

[1]Department of Biomedical Research, Nemours/A.I. duPont Hospital for Children, Wilmington, DE, USA.

[2]Department of Biological Sciences, University of Delaware, Newark, DE, USA.

Correspondence:

Zhengyu Ma, Ph.D.

zma@nemours.org

Supplementary Figures

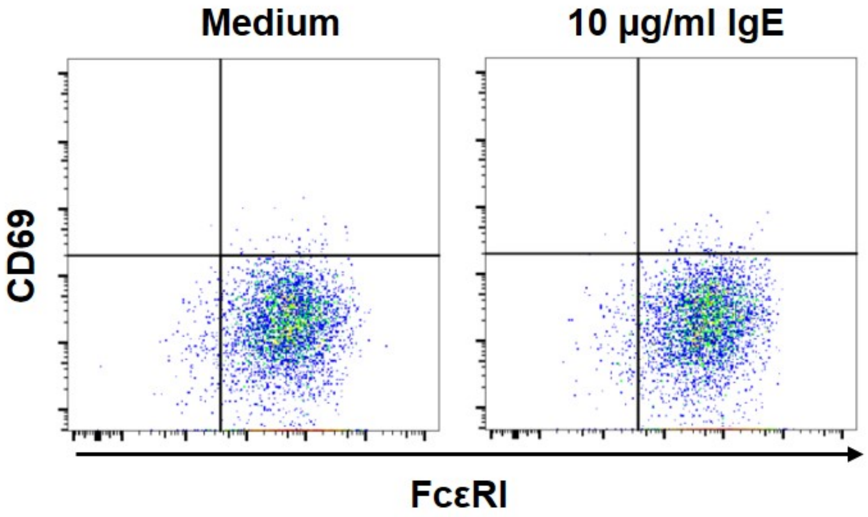

Supplementary Figure 1. Jurkat cells expressing WT FcεRIα-based CAR do not respond to secreted IgE. 0.25 x 10^6 Jurkat cells expressing WT CAR were incubated in complete RPMI medium with or without 10 µg/ml IgE for 5 hrs and stained with anti-CD69-APC antibody.

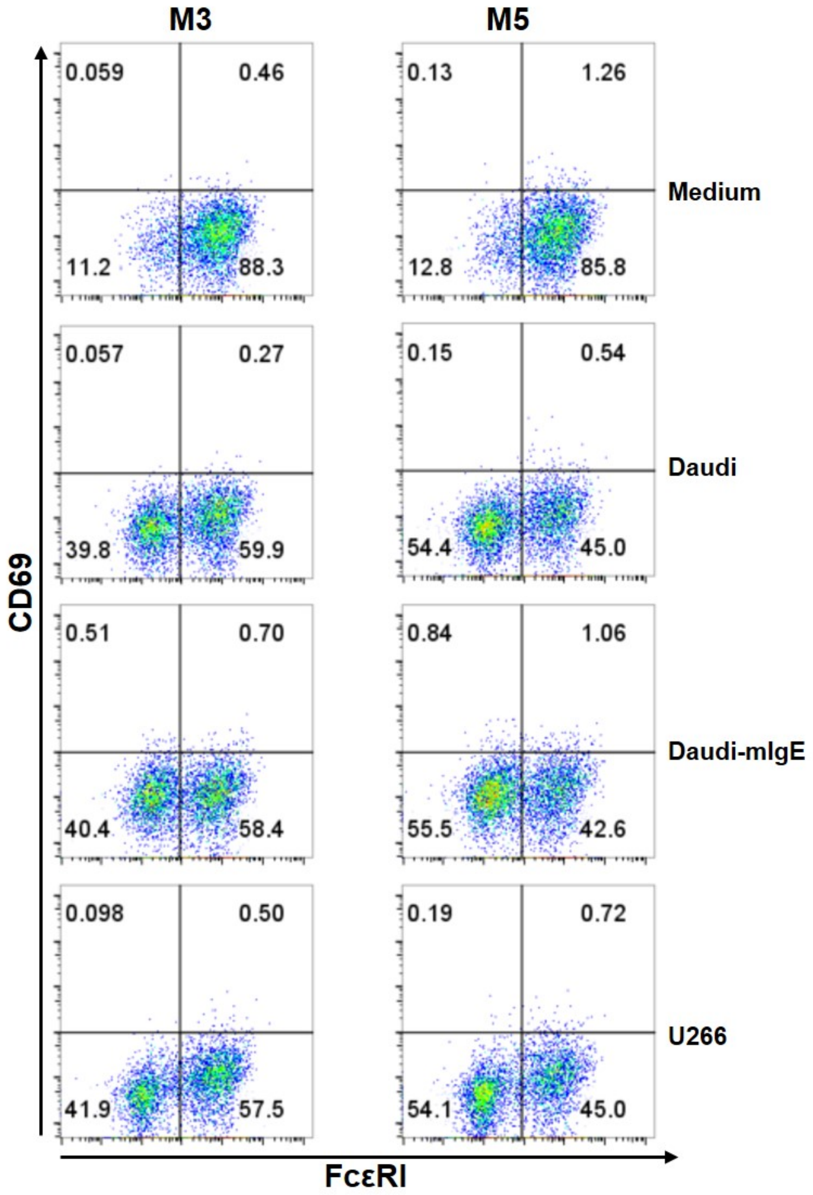

Supplementary Figure 2. Jurkat cells expressing M3 and M5 FcεRIα-based CAR do not respond to U266 or Daudi-mIgE cells. 0.25 x 10^6 Jurkat cells expressing M3 or M5 CAR were incubated in medium or with equal number of Daudi, Daudi-mIgE or U266 cells for 5 hrs and stained with anti-FcεRIα-PE and anti-CD69-APC antibodies.

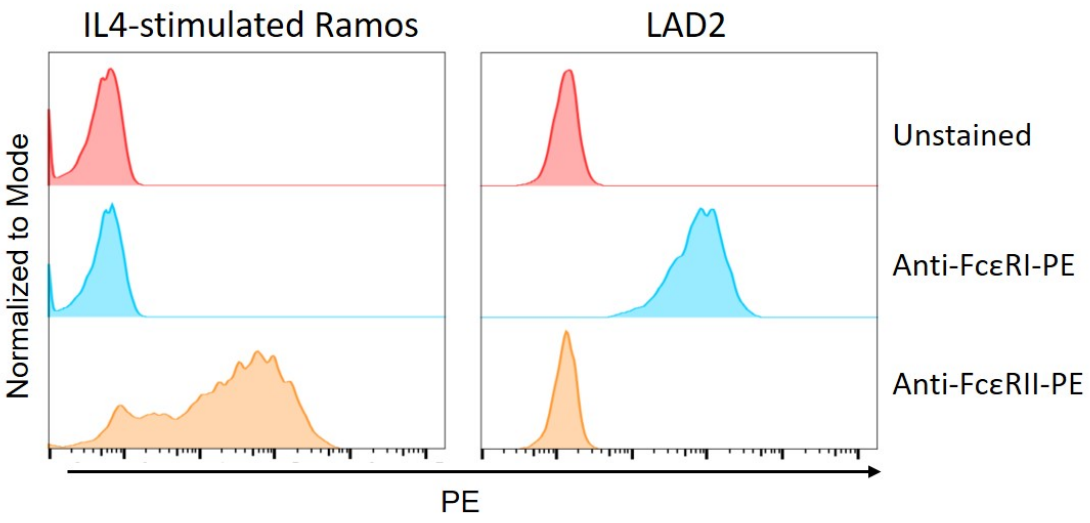

Supplementary Figure 3. FcεRI and FcεRII expression on Ramos and LAD2 cells. To upregulate FcεRII expression, $3x10^4$ Ramos cells were incubated in complete RPMI medium with 20 ng/ml of recombinant human IL4 for 72 hrs. IL4-stimulated Ramos cells and LAD2 cells were stained with PE-labeled anti-FcεRI-PE or anti-FcεRII-PE antibody for flow cytometry.

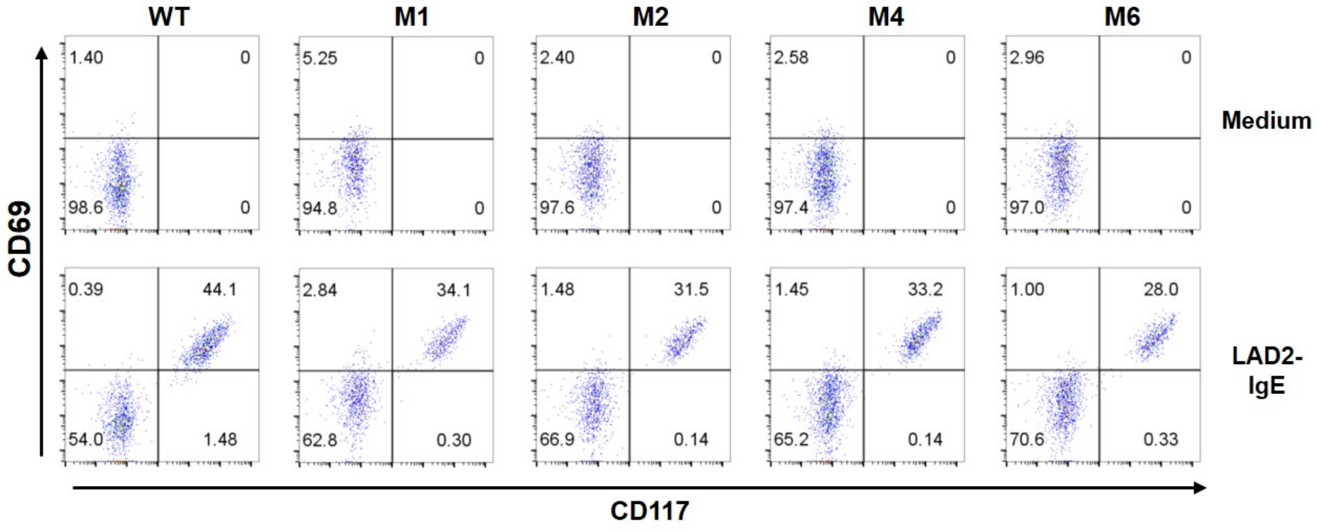

Supplementary Figure 4. FcεRIα-based CARs do not mediate T cell responses to mast cells with free IgE captured through FcεRI. LAD2 cells with IgE bound were used to stimulate CAR^+ Jurkat T cells for 5 hrs. Cells were collected and stained with antibodies for CD69 and CD117. CD117 is expressed only on LAD2 cells and therefore used to distinguish Jurkat and LAD2 cells. Data are representative of two independent experiments.
